# Feasibility and usability of remote transcranial direct current stimulation (tDCS) for self-regulation in children with autism: protocol for a randomized controlled pilot study

**DOI:** 10.1186/s40814-025-01650-4

**Published:** 2025-04-29

**Authors:** Norna Abbo, Trina Mitchell, Seyed Hassan Tonekaboni, Evdokia Anagnostou, Brendan F. Andrade, Kevin Thorpe, Deryk S. Beal

**Affiliations:** 1 Bloorview Research Institute, Holland Bloorview Kids Rehabilitation, Toronto, Canada; 2 Institute of Biomedical Engineering, University of Toronto, Toronto, Canada; 3 Department of Paediatrics, Temerty Faculty of Medicine, University of Toronto, Toronto, Canada; 4 Centre for Addiction and Mental Health, Toronto, Canada; 5 Department of Psychiatry, University of Toronto, Toronto, Canada; 6 Dalla Lana School of Public Health, University of Toronto, Toronto, Canada; 7 Department of Speech Language Pathology, Temerty Faculty of Medicine, University of Toronto, Toronto, Canada

**Keywords:** Neurostimulation, Autism spectrum disorder, Children, Self-regulation, Transcranial direct current stimulation, Feasibility, Pilot study, Usability

## Abstract

**Background:**

Autism spectrum disorder (ASD) is a neurodevelopmental disorder characterized by social communication and self-regulation impairments. Impaired response inhibition and self-regulation in ASD have been shown to be related to abnormal functional network connectivity in the dorsolateral prefrontal cortices (DLPFC). Transcranial direct current stimulation (tDCS) of DLPFC is a safe, tolerable, and precise intervention that has shown promise for the improvement of self-regulatory behavior in ASD. However, clinical translation has been prevented by a lack of effective systematic design, experimental control, and a high participation burden. The proposed protocol aims to evaluate the feasibility and usability of home-based tDCS to promote self-regulation in children with ASD.

**Methods:**

Participants will be randomized into an active or sham tDCS group and will receive 20 min of stimulation 5 days per week for 3 weeks. Participants in the sham group receive a negligible amount of stimulation. Sessions will be virtually supported by the study team. Assessments are taken at baseline, 1-week post-treatment, and 18 weeks post-treatment. These assessments include clinical measures of self-regulation and social communication (participant-, parent-, and clinician-reported), a response inhibition task, and magnetic resonance imaging. Recruitment, retention, and adherence rates will be used to assess the feasibility of the protocol. The usability of the remote tDCS device will be assessed via a usability survey, user interviews, and video analysis of device use.

**Discussion:**

Home-based tDCS may benefit children by providing an efficient, passive, and tolerable treatment that positively impacts function, activities, and participation. This study will identify potential challenges for the clinical translation of this therapy so that home-based tDCS can be positioned for success in healthcare delivery implementation.

**Trial registration:**

ClinicalTrials.gov, NCT06129058. Registered on November 8, 2024.

**Supplementary Information:**

The online version contains supplementary material available at 10.1186/s40814-025-01650-4.

## Background

Autism spectrum disorder (ASD) is a neurodevelopmental disorder that affects 1% of children globally [1], resulting in emotional and economic burdens on families and creating a high demand for intensive resource support in health care systems [2]. ASD encompasses a wide range of symptoms, including cognitive, social communication, and self-regulation challenges. The combination and severity of these symptoms varies across individuals, resulting in significant heterogeneity [3]. Self-regulation is a key mechanism underlying many of the behavioral challenges experienced by individuals with ASD and is defined as the ability to control, monitor, and manage emotions, cognition, and behaviors in a goal-oriented manner [4]. Key components of self-regulation include response inhibition, which is the ability to suppress contextually inappropriate responses, and emotional regulation, which is the ability to modify arousal and reactivity to engage in adaptive behavior [5]. Self-regulation is strongly correlated with the development of social communication skills, mental health outcomes, and academic achievement in children with ASD [6, 7]. Impaired self-regulation is a predictor of quality of life and companionship in adulthood and is associated with increased parental stress, family and peer discord, and risk of social stigmatization [8, 9]. There is a lack of effective interventions for the promotion of self-regulation in children with ASD and behavioral interventions are often time and resource-intensive for families and providers and yield small effect sizes for patient-reported outcomes [10]. Children with ASD, their families, caregivers, and clinicians have identified the urgent need to establish novel, effective interventions that promote self-regulation [10].

Impairments in response inhibition play a prominent role in self-regulation in ASD and the co-morbid disruptive, compulsive, aggressive, and self-injurious behaviors that are associated [11–13]. The neural mechanisms for self-regulation are relatively well established and are defined by fronto-subcortical and fronto-limbic networks [14, 15]. These neural networks are compromised in ASD as characterized by an overgrowth of brain volume in early neurodevelopment, which may lead to local hyperconnectivity in frontal and occipital regions, and long-range hypoconnectivity between frontal regions and other cortical areas [16]. Specifically, the dorsolateral prefrontal cortices (DLPFC) and inferior frontal gyri are commonly reported to have abnormal functional network activity patterns relative to controls that are indicative of poor top-down response inhibition and self-regulation in this population [12, 17].

Transcranial direct current stimulation (tDCS) is a safe, tolerable, and precise intervention that can target these neural networks and has shown promise for the reduction of ASD symptoms. It runs a current through target brain regions via an anode and a cathode. The magnitude of the electric field elicited by tDCS has been shown to be sufficient in modulating neural activity on a cortical network level [18]. This is due to the coupling mechanisms of local endogenous fields, which result in the amplification of the electric field produced by tDCS [19]. Open-label and waitlist-controlled studies have indicated that neuromodulation of prefrontal regions results in improvements in response inhibition on neural signatures (e.g., MER ratio), cognitive tasks (e.g., Go/NoGo), social communication measures, Autism Treatment Evaluation Checklist (ATEC) sociability sub-scores, and parent-reported clinical health outcomes (e.g., reduced compulsive, aggressive, and self-injurious behaviors) [20, 21].

Clinical translation of tDCS has been prevented by a lack of systematic design and experimental control, small and poorly represented samples, inadequate blinding, insufficient follow-up periods, and a paucity of patient-reported outcome measures [22, 23]. Further, the participation burden was extremely high in previous studies examining tDCS, as they required multiple trips to the laboratory. Home-based tDCS has the potential to benefit children with ASD by providing an efficient, passive, accessible, and tolerable treatment that positively impacts function, activities, and participation. However, there is limited research investigating tDCS in this population and no studies examining home-based tDCS specifically. To address this gap, a neurobiologically informed pilot study is needed to determine if it is feasible, tolerable, and acceptable to pursue a full-scale randomized control trial (RCT) to test if home-based tDCS therapy is efficacious for improving self-regulation across multiple levels of integration (cognitive, behavioral, neural) in children with ASD.

## Methods

### Design

This study is a double-blind pilot RCT examining the feasibility of carrying out a full-scale RCT to determine if 15 sessions of home-based tDCS therapy can improve clinical, cognitive, and neural outcomes associated with self-regulation in children with ASD. Measures are taken at baseline (T0), 1-week post-treatment (T1), and 12 weeks post-treatment (T2).

### Participants

#### Recruitment

Children with ASD with self-regulation impairments (*n* = 46) aged 9–18 years will be recruited from the Province of Ontario Neurodevelopmental Disorders Network (POND) (www.pond-network.ca). The lower limit of nine years old was determined based on previous studies investigating the safety and tolerability of non-invasive brain stimulation (NIBS) in children with ASD [24, 25]. POND participants have an existing research-reliable ASD diagnosis based on DSM-V criteria, informed by the gold standard Autism Diagnostic Observation Schedule (ADOS) [22] and the Autism Diagnostic Interview – Revised (ADI-R) [26]. Recruitment flyers will be sent to POND families with study information and contacts. Potential participants will be contacted by a research assistant until the recruitment target is reached.

#### Eligibility

Self-regulation impairment will be defined by a score of four or greater on items 4 (disruptive behavior) or 5 (restricted and repetitive behavior) on the Clinical Global Impressions Severity (CGI-S) administered by a physician during screening [27]. As this is a feasibility study, there will be no intelligence quotient (IQ) cut-off for participation. It is critical that novel treatments for response inhibition and self-regulation be studied in the broader autism community, as children with lower IQs more frequently exhibit social impairments and self-regulation challenges [28, 29], and are not typically candidates for behavioral treatments that require high learning demands and cognitive effort. Children with safety contraindications for tDCS (e.g., seizure disorder), magnetic resonance imaging (MRI) (e.g., metal in situ), or co-morbid neurological disorders (e.g., epilepsy, stroke, brain tumor) will be excluded. Children must not have had any changes to their medications for at least 6 weeks prior to the baseline assessment (T0) and no planned medication changes during the study. Children with prior tDCS experience will be excluded to ensure blinding.

### Consent

Consent will be obtained electronically using the REB-approved documents via REDCap or in person. In-person consent will be conducted by a designated study team member who will complete the capacity assessment and consent discussion. If the participant does not successfully complete the capacity assessment their parent/substitute decision maker will be given the option to sign the parent consent, and the participant will be provided the option to sign the assent form. Electronic consent will follow the same format; however, the designated study team member will complete the capacity assessment and consent discussion, as well as answer any questions, at a scheduled BRI videoconferencing (Zoom) session with the participant and their parent/guardians. Prior to attending the scheduled consent discussion, participants and their families will be provided a read-only copy of the consent document to review together. Non-verbal consent or assent will be documented appropriately as needed.

### Sample size

Pilot RCTs do not test efficacy, and formal sample size calculations are not typically used. However, if too small, a pilot study may underestimate the standard deviation leading to underpowered efficacy RCTs. Sim et al. [30] suggested that the sample size of a pilot study be determined based on the desired level of confidence for the standard deviation of the primary outcome measure in the larger-scale RCT, and the power and significance level anticipated. Additionally, attempts to minimize the total sample size required should be made. Given an expected 10% loss to follow-up, and to maintain equal group sizes, the proposed pilot sample size will be *n* = 46. This is adequate to assess recruitment success and feasibility issues and provides sufficient data for exploratory analysis of the secondary outcomes.

### Randomization and blinding

Participants will be randomized to either the active tDCS or sham tDCS treatment condition in a 1:1 allocation ratio. An independent research assistant will prepare a randomization schedule using random permuted blocks of varying sizes to ensure allocation concealment and will assign participants to active or sham tDCS groups. Block sizes and variations will only be known to the independent research assistant. The allocation sequence will be entered into a password-protected Excel sheet. A separate, blinded member of the study team will enroll participants. Participants, family members, assessors, and investigators will all be blinded to group allocation. The devices will be preprogrammed with codes that correspond to specific stimulation parameters. During each session, participants will be provided with a code aligning with their group assignment to maintain blinding. Unblinding of participants will be permissible if the participant is no longer participating in study procedures and requires information on group allocation for medical purposes. In this case, the principal study investigator will reveal group membership to the participant. To minimize observer bias, the principal investigator will not be involved in data collection or analysis.

### tDCS intervention

Home-based tDCS will be delivered via a Soterix 1 × 1 mini-CT remote tDCS neuromodulation device (Soterix, New York, NY). The device will be equipped with a two-channel Omni-Lateral-Electrode-System (OLE) montage. The OLE montage is specific to left DLPFC targeting, delivering anodal stimulation to the left DLPFC and cathodal stimulation to the right DLPFC [31]. In the standard view of tDCS, anodal stimulation results in an excitatory response in the underlying brain region, and cathodal stimulation results in an inhibitory response [32]. Given that the left DLPFC is typically underactive in children with ASD, anodal stimulation over this area might increase the opportunity for activity to increase to typical levels. Anodal stimulation over left DLPFC has been shown to improve executive functioning in individuals with autism [33–35]. The results of stimulation of the right DLPFC have been mixed, with both anodal and cathodal stimulation showing positive effects on behavior [36]. Neuroimaging studies have also shown both hypo- and hyperactivation in the right DLPFC of children with autism when compared to controls. These findings indicate that modulation of both the left and right DLPFC may contribute to improvements in behavior. The left anodal and right cathodal montage have shown the most promising results in tDCS studies in this population and is the most well-documented [18, 36–40].

Participants in the active tDCS group will receive a total of 20 min of stimulation per session, at a maximum of 2 mA intensity. For the sham tDCS control group, the same device, montage, and protocol will be used, but the device will be programmed for the sham condition. This condition delivers 30 s of stimulation (max. 1.0 mA) at the start and end of the session to mimic the sensation of the active condition. This is the proven gold standard for participant blinding and sham-control [41–43]. The first treatment session will ramp up to 1.0 mA for all participants. Participants in the sham tDCS group who tolerate 1.0 mA will remain at 1.0 mA in each treatment session. Participants who do not tolerate 1.0 mA will have the current adjusted to 0.75 mA and 1.0 mA will be attempted again at the next session. For the active tDCS group, if 1.0 mA in the first treatment session is tolerated, the second treatment session will begin at 1.5 mA. If 1.0 mA is not tolerated, the session will start at 1.0 mA. If 1.5 mA is tolerated throughout the second treatment session, the third treatment session will begin at 2.0 mA. If 1.0 mA is tolerated throughout the second treatment session, the third treatment session will begin at 1.5 mA. For the remainder of sessions, the intensity tolerated in the final 10 min of one treatment session will serve as the starting point in the next treatment session. If a participant is not tolerating a certain intensity at any point during treatment, it will be reduced by 0.25–0.5 mA, and their symptoms will be re-evaluated at the reduced intensity and adjusted accordingly.

**Fig. 1:**
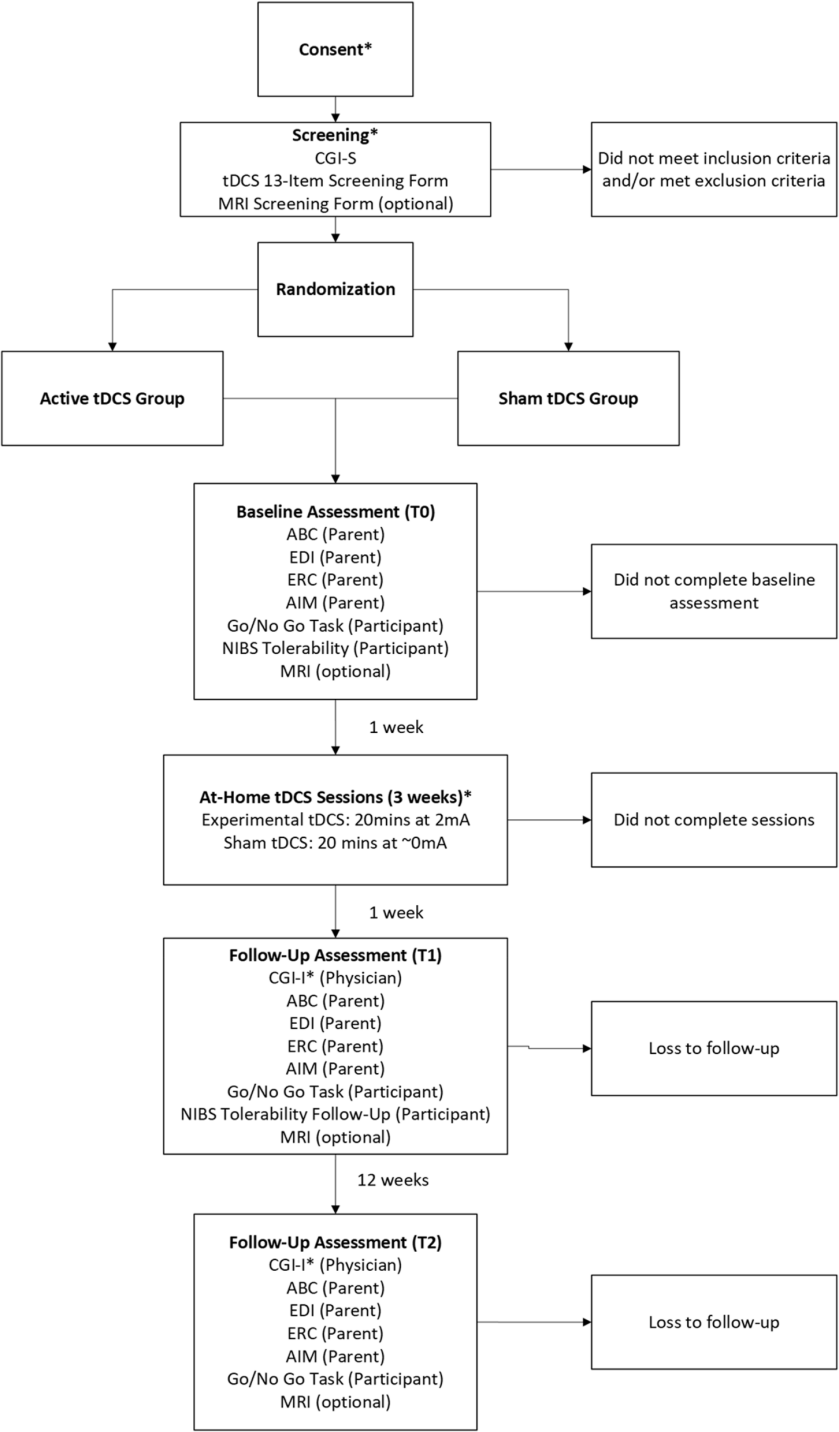
Overview of the study design. (*) designates portions that can be completed virtually

A summary of the study schedule is shown in Fig. 1. All in-person assessments will be conducted at Holland Bloorview Kids Rehabilitation Hospital in Toronto, Canada. The take-home tDCS device will be provided at the first baseline assessment visit (T0). The participant and/or caregiver will be trained on administration and device use by a study team member. The study team member will demonstrate how to use the device on the participant in a step-by-step manner. Following the demonstration, the caregiver or participant will attempt to recreate the demonstration, and the study team member will observe to ensure they are following the steps correctly and assist with any questions or concerns. The following week, participants will begin home-based tDCS treatment sessions. Fifteen sessions will be conducted over 3 weeks (5 days/week, Monday–Friday), with remote support from the study team via videoconferencing [42, 44]. Each session will be 30 min in duration, including a 5-min intake and equipment set-up, 20-min tDCS, and 5-min debrief and observation to ensure there are no unanticipated adverse events [34, 45]. Previous studies have shown that 15 sessions of 20-min tDCS at 2 mA may be effective in this population for a variety of behaviors [18, 46].

### Primary outcome measures

The primary objectives of this study are to assess the feasibility, tolerability, and acceptability of an RCT to test the efficacy of tDCS. Feasibility measures are based on a hypothetical sample size of 100 participants for a full-scale RCT, based on previous neurostimulation clinical trials [47–51].

#### Feasibility

Feasibility measures include recruitment rate, retention rate, adherence rate, and success of randomization and blinding. Recruitment and attrition will be calculated using the electronic audit trail generated by the documentation kept by the independent RA and statistician and research assistant. Adherence will be calculated based on the number of sessions attended and completed by participants who complete all assessments. The success of blinding will be determined based on responses to the questions “What group do you think you were in?” and “How sure are you?”, which will be asked on the follow-up tolerability questionnaire (adapted from Garvey et al., 2001) [52]. Descriptive statistics will be completed to establish if the comparison and intervention groups are similar in age, gender, ASD severity, and clinical symptom severity. The success of randomization will be determined by evaluating group differences in sex distribution and median age. Randomization will be determined to be successful if the differences in sex distribution are within ± 2 participants, and the median age difference is within ± 2 years.

The feasibility of the optional MRI component will be evaluated based on total and successful attempts at completing the MRI protocol. There is a paucity of data on neural outcomes in this population; therefore, any amount of data collected is valuable. Previous MRI studies examining children with ASD include samples between 20 and 50 participants [53–57]. In a hypothetical sample size, *n* = 100 (*n* = 50 per group) for a full-scale RCT, including data from at least 20 individuals in each group would be sufficient to conduct an analysis. Given this, the optional MRI component of this study will be feasible if ≥ 20% of participants in each group have successful MRIs. A successful attempt is one in which a majority of the scan is completed with minimal motion artifact as determined by the MRI technician.

#### Safety and tolerability

At T0 and T1, participants complete a baseline and follow-up tDCS tolerability questionnaire, which assesses their experience with the stimulation and their assumption of group membership. At each tDCS session, a full body 16-item systems questionnaire and the NIBS tolerability questionnaire will be completed by the participant and parent to assess the participant’s tolerability of tDCS and to monitor any side effects or adverse events [58–60]. Adverse event (AE) monitoring will begin at the baseline assessment and continue through the study timeline. All AEs will be evaluated by the study physician for seriousness, severity, expectedness, and relatedness to the intervention, using a validated AE worksheet (see Appendix).

#### Acceptability and device usability

To evaluate acceptability, a treatment satisfaction scale will be administered at T1. This survey asks participants to rate how helpful they found the tDCS to be, and if they would recommend it to others, as well as open-ended questions about how the tDCS experience can be improved [61]. The standard device training will be video-recorded at T0 for usability analysis. A pre-treatment (T0) and post-treatment (T1) usability survey will also be administered to understand any barriers to using the take-home tDCS device (developed based on the system usability scale [62, 63]). The pre-treatment survey will be used to understand the caregiver’s familiarity with technology in a general sense [64]. At T2, a semi-structured interview will be administered to gain a deeper understanding of participants’ experiences with the tDCS device.

### Secondary outcome measures

Secondary objectives include (i) estimating the probable effect of tDCS on clinical, cognitive (Go/NoGo), and neural (fMRI, DTI) self-regulation outcomes; (ii) exploring the factors influencing outcomes; (iii) exploring the factors influencing participation/refusal. Outcomes will be measured at baseline (T0), week 6 (T1), and week 18 (T2) by blind assessors and data analysts. tDCS sessions occur during weeks 1–3.

#### Clinical

The CGI severity [65] and improvement assessments include an interview by the physician with the participant and caregiver. Parent-reported measures include the Aberrant Behaviour Checklist (ABC) [66, 67], Emotional Dysregulation Inventory (EDI) [68, 69], Autism Impact Measure (AIM) [70], and Emotional Regulation Checklist (ERC) [71]. These measures have been validated in youth with ASD and will indicate clinical treatment-related improvement. The ABC has excellent reliability and was specifically designed to measure treatment effects on aberrant behaviors, with extensive application in previous ASD trials [72]. The EDI-reactivity scale measures poorly regulated negative emotions, and the EDI-dysphoria scale measures minimal positive affect and motivation. The AIM measures treatment-related improvements in children with ASD across several ASD symptom domains [70]. The ERC measures emotional regulation and is sensitive to a broad range of regulatory processes [73].

#### Cognitive

The PsyToolkit Go/No Go task will be used to measure response inhibition. Performance on the Go/No Go task is measured by response time and accuracy [74]. This specific Go/No Go task was chosen due to its simplicity and ability to be understood by individuals with a wide range of IQs and autism severity.

#### Neural

At the baseline assessment (T0), participants will undergo optional high-resolution MRI on a Siemens 3 T Prisma MRI. Structural T1-MPRAGE, DTI, and resting-state functional MRI data will be collected at all time points (T0–T2). Acquisition of neuroimaging data will allow for the investigation of changes at immediate post-treatment (T1) and maintenance at long-term follow-up (T2).

At the baseline assessment, participants will undergo an optional high-resolution MRI on a Siemens 3 T Prisma MRI. Structural (T1 and diffusion tensor imaging) and resting-state functional MRI data will be collected at all timepoints. Acquisition of neuroimaging data will allow for the investigation of changes at immediate post-treatment and maintenance at long-term follow-up.

### Data safety and sharing

#### Data management

All research data will be de-identified. All participants will be given a unique code that will be linked to their personal information accessible only by the research team. All identifiable information will be kept confidential and stored and locked in a secure place that only the study staff will be able to access. Electronic files will be stored securely on hospital or institutionally approved networks or securely on any hospital or institutionally approved portable electronic devices. Research results will be shared through journal publications, academic conferences, and knowledge translation activities at Holland Bloorview. Research assistants and the principal study investigator will have access to the final participant dataset. Protocol amendments will be submitted directly to the REB for review. Once accepted, changes will be communicated to participants directly by the study members.

#### Data safety monitoring

A data safety monitoring committee will meet regularly to review all adverse event reports and study progress every 6 months. The committee will be chaired by a physician and membership will include a scientist, nurse, and lay person/family leader. All adverse events (AE) will be monitored by a safety monitoring committee (SMC) acting independently of the investigators. This SMC will consist of a pediatrician, a methodologist/researcher, and a nurse. This committee will examine all AE reports to ensure that harm is not occurring. The SMC will decide whether the trial needs to be stopped based on rules that they will set at their first meeting in accordance with the documented risks of tDCS. Participant symptoms and safety will be tracked during and after each tDCS session, and all adverse events reported via an REB-approved standard process to an independent in-house safety monitoring committee. Stopping rules for the study based on adverse events will be in place as determined by the SMC. Participants will also be given an emergency number that is monitored by a physician on the study team.

#### Stopping guidelines

At each tDCS session, the study personnel will monitor the participant’s symptoms by completing the systems questionnaire and the NIBS safety and tolerability form. The study personnel will monitor any symptoms (e.g., headache) after the tDCS by having them rate any symptoms on a scale of 1–5. If any symptoms last longer than 15 min graded at 4 or greater, even upon discontinuing tDCS, stopping protocols will be initiated. In the event of a new onset of neurological symptoms, such as severe headache or nausea, the session will be stopped immediately, and the family will be advised to seek immediate medical attention.

If the study personnel, study investigator, or child/parent has any concerns about starting or continuing with a treatment session, the session will be put on hold. Study personnel made aware of any concerns will report them to the study investigator and study physician. The study physician will determine whether the complaint warrants medical attention or adjustment of the treatment. If the severity does not warrant medical treatment, several monitoring and prophylactic measures will be taken. They will be informed that they will be called by our study personnel the next day to get a progress update. Parent concerns at that point will be directed to their family physician to be checked.

### Data analysis

#### Primary measures analysis

##### Feasibility

Feasibility measures will be analyzed as per the progression criteria outlined in Table 1. These criteria were determined based on recommendations by Avery et al. to assess whether to proceed with a full-scale RCT [75]. Associations between participant characteristics (e.g., age, IQ, travel distance) and ability to complete all trial tasks will be examined to inform the planning of a large-scale RCT. *T*-tests will be conducted for associations with continuous variables (e.g., IQ, age, travel distance). Chi-squared tests will be conducted for associations with categorical variables (e.g., gender).

**Table 1:** Progression criteria for feasibility metrics

	**GO–proceed with RCT**	**AMEND–proceed with changes**	**STOP–do not proceed unless changes are possible**
Recruitment target	*n* = 46	*n* = 36–45	*n* < 36
Adherence	≥ 90% of tDCS sessions are completed by participants who complete all assessments	70–89% of tDCS sessions are completed by participants who complete all assessments	< 70% of tDCS sessions are completed by participants who complete all assessments
Participant retention	≥ 90% retention	80–89% retention	< 80% retention
Optional MRI target	≥ 20% of participants have successful MRIs in each group	10–19% of participants have successful MRIs in each group	< 10% of participants have successful MRIs in each group

##### Safety and tolerability

Safety will be assessed by the occurrence of side effects as reported on the systems and NIBS tolerability questionnaires, in addition to the instances and severity of AEs. A correlational analysis will be conducted between the occurrence of side effects and feasibility measures to assess the association between these factors.

##### Device usability and protocol acceptability

Device usability is described by three measures: effectiveness, efficiency, and satisfaction. Effectiveness is determined by the degree of task completion and the total number of errors per task, which will be identified in the training video recordings. Task completion will be coded as follows: (1) completed independently by the user, (2) completed with minor help from the trainer, (3) failed to complete even with help from the trainer. Errors are coded when the user makes mistakes that prevent the continuation of the task without help from the trainer. Efficiency is measured by calculating the average time taken for each task across participants. A table of the tasks and criteria for completion is shown in Table 2. The post-treatment usability survey will assess participants’ satisfaction with the use of the device. The survey includes three sections: “device”, “headband”, and “virtual delivery.” Answers to positive statements are scored positively, and answers to negative statements are scored reversely. The highest possible raw score for each section is 55, 15, and 25, respectively. The raw scores will be converted to a standardized total for each section. A score of 80 or higher will be considered “excellent” as per standard SUS scoring. Users will be given the opportunity to include comments and feedback on each section.

**Table 2:** Task analysis and completion criteria for tDCS device use

Task	Task completion criteria
**Place batteries into device**	Correct orientation of batteries
**Put sponges on headband**	Correct orientation of sponges
	Securely attached to headband
**Plug headband into device**	Correct color wire into the correct color port (red-red, black-black)
**Turn on device**	Pressed correct button
**Initiate “stimulation” setting**	Pressed correct button
**Place headband on participant**	Correct orientation of headband
	Remove hair underneath sponges
	Contact quality: Good

The pre-treatment usability survey provides information on the general technological knowledge and ability of the person administering the tDCS. This score will be correlated with the score on the post-treatment survey to provide insight into how varying levels of technological knowledge may impact the usability experience of the tDCS device.

#### Secondary measures analysis

A modified intent-to-treat analysis will be used for secondary data. Analyses of secondary measures will only include participants who have provided data at baseline and at least one follow-up timepoint.

##### Clinical and cognitive

For clinical assessments and the Go/No Go task, between-group differences over time will be examined using a linear mixed-effects model approach. Follow-up scores for each outcome will be included as dependent variables, and baseline and treatment allocation values will be included as fixed-effect predictors. Time points will be included as a fixed factor. To account for the correlation between repeated measurements from the same participant, a random intercept will be included at the participant level.

##### Neural

Structural and functional neuroimaging changes between timepoints T0, T1, and T2 will be compared between groups, and associations between baseline neuroimaging and treatment outcomes will be explored. Freesurfer (https://surfer.nmr.mgh.harvard.edu/) will be used to obtain cortical thickness from structural MRI data. MRtrix3 software will be used to process diffusion imaging data to determine changes in white matter tracts [76]. Blood-oxygen-level-dependent (BOLD) signal of resting-state functional MRI will be analyzed using the fMRIPrep pipeline. All results will be corrected for multiple comparisons using the false discovery rate (FDR) method (*P*_FDR_ < 0.05). Changes in network activity will be analyzed using the left DLPFC as a seed.

## Discussion

To establish the clinical effectiveness of home-based tDCS for self-regulation in ASD, a well-designed RCT is needed. However, efficacy RCTs are highly resource intensive [77]. This pilot RCT will provide critical insights that position an efficacy RCT for success and ensure that resources are invested in trials likely to generate clinically meaningful results [78].

The design of the current study is novel and addresses important limitations of previous studies. There is relatively limited research into the effects of tDCS on ASD in children with the findings reported in the existing literature being inconsistent and unconvincing. Several reviews and meta-analyses have concurred that there is a need for well-designed randomized, double-blinded studies with long-term follow-up measurements to increase the quality of evidence for tDCS [18, 36, 39, 46, 79]. Most existing studies are open-label or case reports, and fewer than half of the clinical trials utilized a sham-control group [36, 39]. Current research is also lacking in longitudinal data, which is important in evaluating the long-term safety and efficacy of tDCS. By including both 1-week and 18-week follow-up timepoints, this protocol allows for the evaluation of whether it is feasible to capture this longitudinal data.

This protocol minimizes many of the biases that are present in earlier tDCS studies. The use of randomization, double-blinding, and sham-control reduces performance and selection biases. While previous literature excludes individuals with low IQs [39], the lack of IQ cutoff in this study further reduces the barriers to participation and allows tDCS to be explored in a broader population. Additionally, the heterogeneous nature of neurodevelopmental disorders presents several challenges, particularly due to the common presence of comorbidities. By focusing on a subsample of the ASD population with self-regulatory issues, a common comorbidity is identified, addressing the potential bias of a non-homogeneous sample.

The secondary outcome measures of this study provide a comprehensive assessment of self-regulation and include measures that independently examine specific components of self-regulation (emotional regulation and response inhibition). This is necessary to capture the heterogeneity of behaviors in the ASD population and to determine which clinical measures are sensitive to treatment-related changes. Additionally, very few tDCS studies of self-regulation in ASD have examined neural changes and none have employed quantitative MRI techniques. Existing research has relied on caregiver and participant reports, without including any form of clinician assessment [39]. The inclusion of the CGI assessment in this study, in addition to the randomization and double-blinding, limits the susceptibility of the results to placebo effects.

There is a clear need for accessible and cost-effective treatment options targeting self-regulation in children with ASD. However, there are currently no published studies that investigate home-based tDCS for ASD. The home-based approach has the potential to significantly reduce the burden on families and caregivers and overcome barriers to participation.

## Supplementary Information

Additional file 1: Consent Form

Additional file 2: Usability Demographics Survey

Additional file 3: Usability Survey

Additional file 4: Usability Semi-Structured Interview Questions

Additional file 5: Adverse Event Worksheet

## Data Availability

Not applicable.

## Abbreviations

ASD: Autism spectrum disorder
DLPFC: Dorsolateral prefrontal cortex
tDCS: Transcranial direct current stimulation
ATEC: Autism Treatment Evaluation Checklist
POND: Province of Ontario Neurodevelopmental Disorders
ADI-R: Autism Diagnostic Interview – Revised
CGI-S: Clinical Global Impressions Scale – Severity
IQ: Intelligence quotient
MRI: Magnetic resonance imaging
RCT: Randomized controlled trial
OLE: Omni-lateral electrode
AE: Adverse event
ABC: Aberrant Behavior Checklist
EDI: Emotional Dysregulation Inventory
AIM: Autism Impact Measure
ERC: Emotional Regulation Checklist
SUS: Systems Usability Scale
FDR: False discovery rate
BOLD: Blood-oxygen-level-dependent
NIBS: Non-invasive brain stimulation
