# Supplementary material for: Feasibility and usability of remote transcranial direct current stimulation (tDCS) for self-regulation in children with autism: protocol for a randomized controlled pilot study
Source: Pilot Feasibility Stud. 2025 Apr 29;11:57. doi: 10.1186/s40814-025-01650-4 (PMC12039062; doi:10.1186/s40814-025-01650-4)
Supplement: Supplementary file 2 — Additional file 2: Usability Demographics Survey [file 40814_2025_1650_MOESM2_ESM.docx]

## Usability Demographics Survey
