# Supplementary material for: Feasibility and usability of remote transcranial direct current stimulation (tDCS) for self-regulation in children with autism: protocol for a randomized controlled pilot study
Source: Pilot Feasibility Stud. 2025 Apr 29;11:57. doi: 10.1186/s40814-025-01650-4 (PMC12039062; doi:10.1186/s40814-025-01650-4)
Supplement: Supplementary file 4 — Additional file 4: Usability Semi-Structured Interview Questions [file 40814_2025_1650_MOESM4_ESM.docx]

## Usability Semi-Structured Interview Questions

How was your experience using the tDCS device?

- What did you like about the device? What didn’t you like?
- Was the training sufficient?
- What would have made using the device easier?

How was your experience using the tDCS headband?

- What did you like about the headband? What didn’t you like?
- Was the headband easy to put on?
- Did (participant) have any issues with the headband?
- Did the headband seem comfortable to (participant)?

How was your experience using the virtual support?

- What did you like about the virtual support? What didn’t you like?
- Was the virtual support necessary? Would you have been able to do the sessions on your own if you had access to the device codes?
- Where did you typically have sessions?

Is there anything else you’d like to share about your experience with tDCS?

- Did you have any problems maintaining the device?
- Were the materials difficult to clean?
- Were the materials difficult to store?
